# Oral Rehabilitation of an Osteopetrosis Patient with Osteomyelitis

**DOI:** 10.1155/2016/6930567

**Published:** 2016-04-11

**Authors:** Tamer Celakil, Merve Dogan, Bilge Gokcen Rohlig, Gulumser Evlioglu, Haluk Keskin

**Affiliations:** Department of Prosthodontics, Faculty of Dentistry, Istanbul University, 34093 Istanbul, Turkey

## Abstract

Osteopetrosis is a congenital disorder characterized by increasing osteoclastic function resulting in osteomyelitis in the jaws. Orofacial findings in osteopetrosis patients are unerupted, malformed, or delayed teeth and many dental caries due to vulnerable enamel and dentin and osteomyelitis. Many reports have described that maxilla is an uncommon site of occurrence for osteomyelitis due to cortical bone morphology and collateral circulation. This report aims to discuss clinical features and prosthodontic management of a patient with clinical features of adult form of osteopetrosis and osteomyelitis in both jaws. The patient has reported better masticatory and speech efficiency with removable dentures in maxillary and mandibular jaw and also self-esteem improvement and family interaction.

## 1. Introduction

Osteopetrosis (OP; Albers-Schönberg disease, marble bone disease, generalized congenital osteosclerosis, ivory bones, and osteosclerosis fragilis generalista) is a hereditary disorder characterized by defective osteoclastic function and impaired bone resorption resulting in dense bone [1–4]. OP is stated in an autosomal recessive form (ARO; Infantile Malignant OP) or autosomal dominant form (ADO; Adult Benign OP) [1, 4]. The estimated prevalence of ARO is approximately 1 in 300000 births, and clinical manifestations are generally hematologic, neurologic, and skeletal abnormalities such as fragile bones, anemia, thrombocytopenia, and impaired vision and hearing [5–8]. Furthermore, clinical manifestations of ADO are primarily skeletal manifestations and the incidence of ADO is 1 in 20000 births [6, 9].

Some studies reported that ADO gene is located in chromosome 1p21 [10]. The pathogenesis of ADO involves diminished osteoclast-mediated skeletal resorption. Bone is not resorbed although the number of osteoclasts is often increased. This defective osteoclastic bone resorption may lead to osteosclerosis [11]. Radiological changes of osteosclerosis, such as “bone within a bone” appearance and transverse radiolucent bands, are typical findings of ADO [5, 12, 13].

Orofacial examination in ADO patients generally results in similar findings such as unerupted, malformed, or delayed teeth and many dental caries due to vulnerable enamel and dentin. Furthermore, osteomyelitis (OM) is the most severe complication and orofacial finding of ADO [11].

OM is an infection of bone, often caused by bacteria, that confines to the medullary cavity [14]. Poor vascular circulation on ADO may lead to OM in jaws after dental extractions [11]. Therefore, infection control before and after dental treatment is of vital importance. Once infection and OM are observed in patients with OP, they can be intractable because of poor-wound healing ability [1, 15]. OM in OP patients generally occurs in mandibular jaw. OM of the maxilla is infrequent due to the cortical bone morphology and collateral circulation [16].

Most of the previous studies have presented surgical procedures of ARO and ADO, fewer reports are available from a prosthetic aspect, and prosthetic management of ADO is unclear [1–4, 6, 7, 17–22]. This clinical report presents the prosthetic rehabilitation of an ADO patient with OM in both jaws. There is no published report on prosthetic management in ADO with OM in both jaws.

## 2. Case Presentation

**Figure 1:**
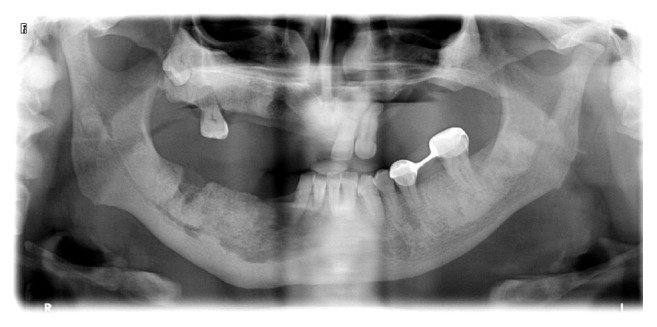
Panoramic radiography of the patient before extraction of the left maxillary central and lateral incisor in 2011.

**Figure 2:**
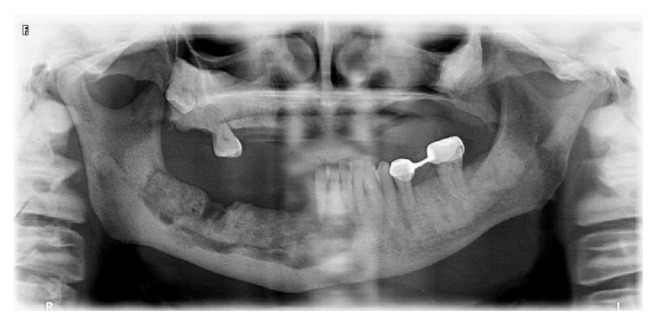
Panoramic radiograph showing osteomyelitis in the right mandibula due to extraction of the right mandibular second molar tooth.

**Figure 3:**
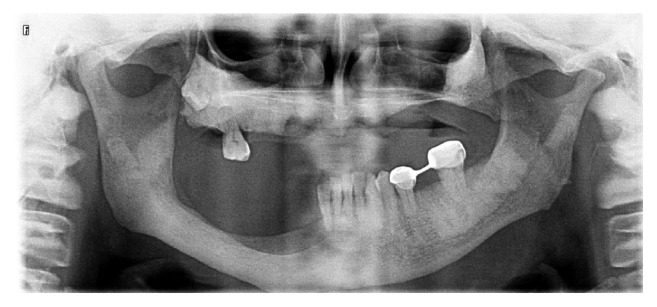
Postoperative panoramic radiography after bony sequestra removed in 2013.

**Figure 4:**
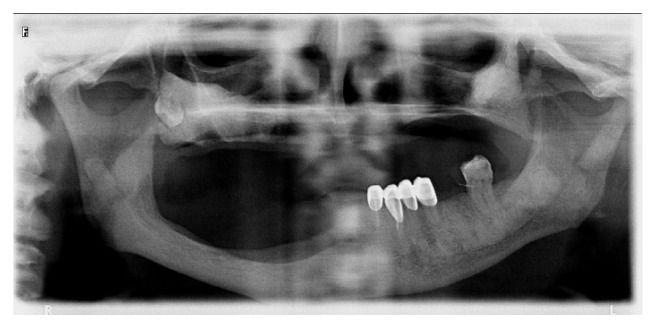
Panoramic radiography view of patient before rehabilitation with prostheses in 2014.

A 48-year-old man suffered from ADO without prior family history of the disease and complained of deficiencies in speaking, swallowing, and mastication due to acquired maxillary and mandibular bone defects. He had been first diagnosed with a right femoral fracture in the 2000s. The patient had purulent drainage in the maxilla in 2003 and he informed no treatment performed in this clinical condition. He underwent surgical procedures, including the extraction of the left maxillary central and lateral incisor and right mandibular second molar tooth under local anesthesia in 2011 (Figures 1 and 2) and the sequestrectomy of right mandible under general anesthesia in 2013 (Figure 3). The patient was referred to the Department of Prosthodontics, Faculty of Dentistry, Istanbul University, for dental rehabilitation in 2014 (Figure 4).

**Figure 5:**
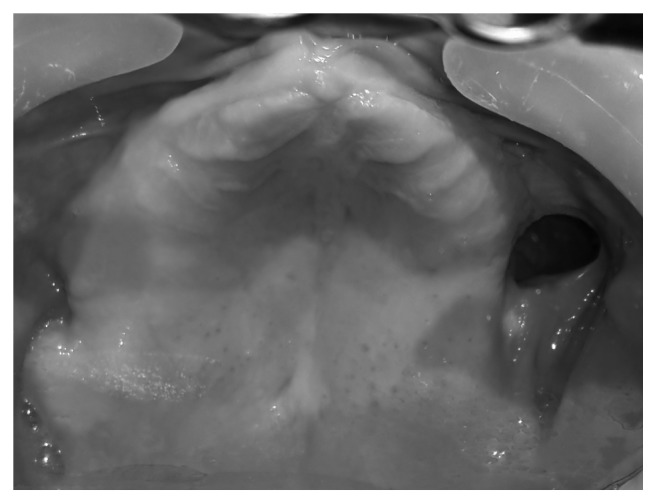
Intraoral photograph showing oronasal fistula in maxilla.

**Figure 6:**
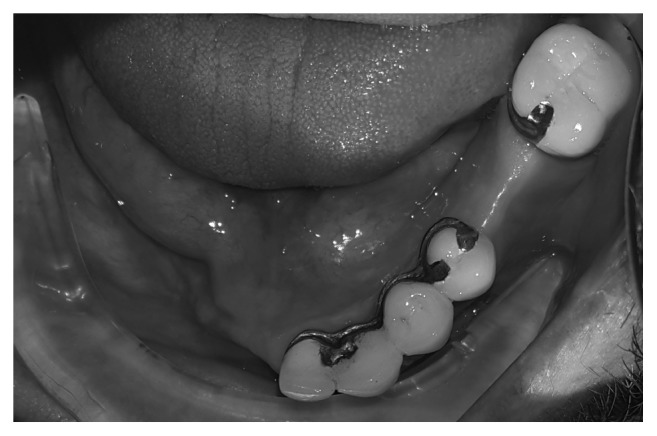
Occlusal surface of fixed prostheses and right mandibular defect due to sequestrectomy.

At radiographic and oral examination, an unerupted third maxillary molar tooth and extensive bone defects in both jaws without any sign of sequestrum were diagnosed. Extraction of the right maxillary first molar, mandibular central incisors, and right mandibular lateral incisor was planned due to severe periodontal disease. Several treatment options were considered and the patient did not agree to any surgical treatment, so the decision was made to replace missing teeth and separation of nasal cavity from oral cavity with removable dentures in maxillary and mandibular jaw (Figures 5 and 6).

**Figure 7:**
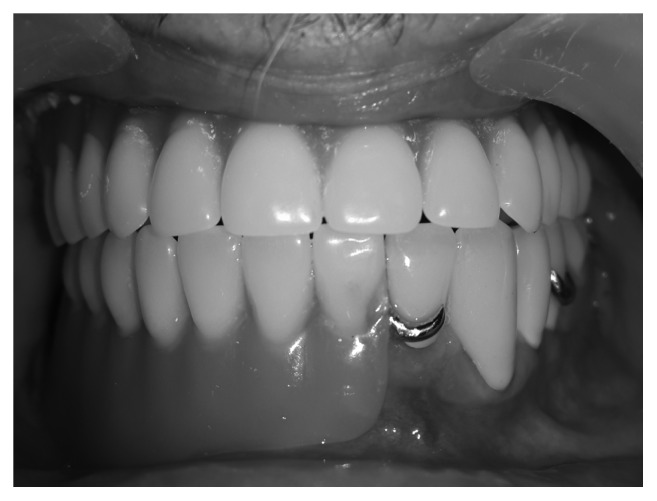
Frontal view with definitive prostheses in maxilla and mandibula.

Antibiotics were provided for prophylaxis to prevent the infection of the bone due to extraction of the teeth. Tooth extractions and preparations were performed under local anesthesia, and the irreversible hydrocolloid impression material was used for making the preliminary impression and fabricating acrylic tray. Adhesive was applied to the tray and condensation silicone (Zetaplus System; Zhermack SpA, Badia Polesine, Italy) was loaded into the tray for master impression. Master casts were fabricated and maxillary master cast was mounted to the semiadjustable articulator (Artex CT; Amann Girrbach AG, Koblach, Austria) to locate the condylar (hinge) axis and mandibular master cast was than mounted to the articulator in centric relation. A maxillary obturator and a mandibular resection prosthesis with a fully balanced occlusion were fabricated. A permanent resilient liner was used to increase the comfort of the affected soft and hard tissues in the maxilla (Figure 7). Determination of the occlusal plane and occlusal adjustment was important for obtaining aesthetic and comfortable results.

**Figure 8:**
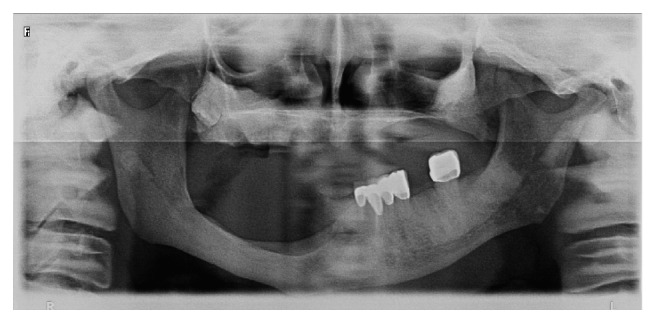
Panoramic radiography of patient at 1-year follow-up visit in 2015.

The patient was satisfied with the aesthetic results and function. The patient has been followed up for 1 year and an oral hygiene program on a 1-month recall schedule was applied (Figure 8). At 1-year follow-up visit, the patient has reported better masticatory and speech efficiency, and also self-esteem improvement and family interaction.

## 3. Discussion 

OP is a hereditary disease characterized by osteosclerosis and OM [22]. OM in OP patients should be treat carefully because OM may lead to some problems such as insufficient function, phonation, and esthetics. This clinical report demonstrates prosthetic treatment of an ADO patient with OM in both jaws.

Gene mutations such as mutations in the ClCN7 genes can be responsible for osteosclerosis and these mutations are generally identified by presence of unerupted tooth [22–24]. Radiological examinations and clinical manifestations are generally adequate in ADO and performing a genetic study is unnecessary to confirm the disease [19, 25]. Some researchers state that conventional radiography and conventional computed tomography (CT), cone-beam computed tomography (CBCT), and magnetic resonance imaging (MRI) may evaluate OM in OP patients. Although some studies have reported that CT is more useful to diagnose OM and evaluate maxillofacial infections [14, 26–29], in this situation, the authors preferred to make the decision for the treatment plan on panoramic radiographs, because the patient was referred to our clinic after his control in Department of Oral Diagnosis and Radiology and he was reported to be infection-free. Differential diagnoses of OP are pycnodysostosis, craniometaphyseal dysplasia, endosteal hyperostosis, diaphyseal dysplasia, melorheostosis, osteosclerosis of fluoride poisoning, and osteopathia striata [11, 19]. After diagnostic analysis, the treatment for associated OM includes antibiotherapy and surgical procedures such as surgical removal of necrotic zone and soft tissue therapy [16, 19, 30]. OP patients generally remain asymptomatic and have a normal life expectancy after antibiotherapy [19].

OP associated with OM of the maxilla is a rare situation for reasons already noticed [6, 19]. The few reported patients in literature confirm the rarity of this condition [19]. In contrast, OM of the maxilla and mandibula was seen in this patient. Surgical interventions such as sequestrectomy, tooth extraction, and free bone grafting should be approached with caution due to the compromised blood circulation, and oral rehabilitation with obturator prostheses in the maxilla is the favored method of filling the defect [1, 19, 31, 32]. However, Naval et al. [33] reported the first patient of OP treated with dental implants and described the protocol used to treat OM that developed after failure of one implant. Prosthetic and surgical interventions should be based on clinical judgment, depending on the presenting conditions and patient needs [1].

This clinical report suggests that surgical therapy modalities such as dental extractions in patients with OP may lead to sclerotic bone areas result in OM in the mandibula and oroantral communication in the maxilla.

A patient with OP and OM in both jaws was treated prosthetically and they fulfilled the requirements of the patient. Prosthetic rehabilitation included the separation of nasal cavity from oral cavity with obturator prosthesis in maxilla and the replacement of missing teeth with removable prosthesis in the mandibula. Prosthodontists should be prepared against maxillary defects that may occur depending on the OM in maxillary jaw and knowledgeable about the rules of obturator prosthesis treatment option.
